# Opportunities and challenges of integrating digital health into medical education curricula: A scoping review

**DOI:** 10.21203/rs.3.rs-6254999/v1

**Published:** 2025-03-25

**Authors:** Wilson Tumuhimbise, Stefanie Theuring, Esther C Atukunda, R Godfrey Mugyenyi, Doreen Babirye, Fred Kaggwa, Rogers Mwavu, Kizza Gerald, Rebecca Nuwematsiko, Irene Wanyana, Daniel Atwine, Twinamasiko Nelson, John Paul Bagala, Richard Mugahi, Geoffrey Namara, Joseph Ngonzi, Rhoda Wanyenze, Juliet N Sekandi, Angella Musiimenta

**Affiliations:** Faculty of Computing and Informatics, Mbarara University of Science and Technology, Mbarara Uganda; African Digital Health Research Advancement Center, Entebbe, Uganda; Institute of International Health, Charité-Universitätsmedizin Berlin, corporate member of Freie Universität Berlin and Humboldt- Universität zu Berlin, Germany; Faculty of Medicine, Mbarara University of Science and Technology, Mbarara Uganda; Faculty of Medicine, Mbarara University of Science and Technology, Mbarara Uganda; Entebbe Regional Referral Hospital, Entebbe, Uganda; Faculty of Computing and Informatics, Mbarara University of Science and Technology, Mbarara Uganda; Faculty of Computing and Informatics, Mbarara University of Science and Technology, Mbarara Uganda; Faculty of Computing and Informatics, Mbarara University of Science and Technology, Mbarara Uganda; School of Public Health, Makerere University, Kampala, Uganda; School of Public Health, Makerere University, Kampala, Uganda; Soar Research Foundation, Mbarara, Uganda; Medical Research Council/UVRI&LSHTM Research Unit, Entebbe, Uganda; Ministry of Health, Kampala, Uganda; Ministry of Health, Kampala, Uganda; WHO Hub for Pandemic and Epidemic Intelligence, Berlin Germany; Faculty of Medicine, Mbarara University of Science and Technology, Mbarara Uganda; School of Public Health, Makerere University, Kampala, Uganda; Global Health Institute, University of Georgia, Georgia, USA; Faculty of Computing and Informatics, Mbarara University of Science and Technology, Mbarara Uganda; Angels Compassion Research and Development Initiative, Mbarara Uganda

**Keywords:** digital health, medical curricula, integration, opportunities, challenges

## Abstract

**Background:**

The global strategy for digital health advocates digital health literacy in formal education and training curricula for all health professionals. However, little is known about the opportunities and challenges of integrating digital health into medical training curricula.

**Methods:**

Guided by Arksey and O’Malley’s scoping review methodology, we searched the PubMed, Google Scholar, and ScienceDirect scholarly databases for peer-reviewed articles published between 2014 and 2024. Data extraction was guided by the consolidated framework for implementation research.

**Results:**

Thirty studies met the inclusion criteria and were analyzed. The opportunities identified include the need for healthcare digitalization, reshaping the future daily work of healthcare professionals, decreasing students’ doubts about digital health and increasing the quality of patients’ care. On the other hand, a lack of infrastructure and educational materials, the dense nature of the existing curriculum, and bureaucratic tendencies were identified as challenges. The provision of consolidated funds and the establishment of dedicated digital health infrastructure, starting with elective and audited modular approaches, raising awareness, and educating stakeholders, emerged as implementation strategies for mitigating these challenges.

**Conclusion:**

Global progress toward integrating digital health literacy in formal medical training curricula remains slow. There is a need for concerted efforts and political commitment to offer guidance and moral and financial support for this integration.

## Background

In 2020, the World Health Assembly endorsed the global strategy of digital health 2020–2025 aimed at improving and accelerating the development and adoption of appropriate person-centric digital health solutions (WHO, 2021). In its efforts to strengthen digital health governance among member states, the WHO digital health agenda highlights the need for an integrated strategy for the sustainable adoption of digital health technologies, training institutions to establish and expand digital health literacy life-long learning opportunities and including them in the education and training curricula of all health professionals and allied health workers to prepare them to deploy and use digital health tools in their daily work (WHO, 2021, Brown and Bewick, 2023).

Healthcare is increasingly becoming digitalized, offering promise for innovative and improved-quality care (Butcher and Hussain, 2022). However, the lack of knowledge and skills to use digital health technologies, negative attitudes, technology anxiety, and scepticism among healthcare workers inhibit adoption and implementation in clinical settings (Frishammar et al., 2023, Machleid et al., 2020, Edo et al., 2023, Ross et al., 2016, HJN, 2023). Intentional training and educational programs for health care workers who play a critical role in the implementation of digital technologies must be prioritized for training if better implementation outcomes are to be realized (Borges do Nascimento and Abdulazeem, 2023). (Khurana et al., 2022). There is a need for mechanisms to increase the capacity of students/future clinicians in the practical use of these technologies as early as possible (Chandrashekar, 2019) by integrating digital health into their curriculum to churn digitally competent future medical workers (Seemann et al., 2023).

The introduction of digital health-specific postgraduate programs aimed at producing graduates with skills and competencies in the application of ICT to improve health practice has been in place for decades. Although this has reshaped digital health knowledge among the few who undertake those programs, a large portion of students who do not take health informatics as a course at the postgraduate level are left with an inadequate understanding of digital health and a lack of preparedness to use it (Kleib et al., 2021). Therefore, despite the availability of these postgraduate digital health courses, opportunities remain skewed toward those with prior qualifications, understanding, and interest (Utukuri et al., 2022).

Digital health remains insufficiently represented in medical education, although it is projected to influence the everyday work of physicians in the next five years (Seemann et al., 2023). Its integration in medical training globally is still in its infancy, but this is understandable given that efforts toward digitizing health care are gaining momentum recently. For example, in Germany in 2019, the deans of 25 European medical schools agreed to rapidly implement digital health education in their medical schools’ curricula (European Deans’ Meeting, 2019). Since then, many studies have explored the integration of digital health teaching in medical curricula (Gillissen et al., 2022, Machleid et al., 2020, Nitsche et al., 2023, Poncette et al., 2020, Vossen et al., 2020) in Europe. A commentary by Aungst and Patel has shown how there is a lack of formal integration of digital health in the medical curriculum of several institutions in the U.S. The majority of these offer certificates in digital health-related courses for those with an interest in the digital health field while leaving out the remaining students who would have benefited from integrating their knowledge with digital health (Aungst and Patel, 2020). Another review by Tudor Car and colleagues documented digital health topics related to courses for medical students, curriculum design, assessment, and evaluation, and challenges related to course development (Tudor Car et al., 2021). Despite this progress, uptake is not guaranteed (Bauer and Kirchner, 2020) if contextual barriers are not systematically identified and if opportunities are leveraged to facilitate increased uptake. This can occur through the implementation science lens, which involves identifying barriers and enablers involved in the uptake of interventions across multiple contextual levels while developing potential strategies for addressing the identified barriers and leveraging the enablers for increased intervention uptake (Bauer and Kirchner, 2020). There is an evident dearth of literature that utilizes implementation science approaches to identify opportunities and challenges in integrating digital health into medical education curricula. Given that many institutions and nations are gearing up for the WHO 2020–2025 digital health agenda, it is paramount to understand challenges that might hinder integration and opportunities to leverage for better outcomes. In this review, we use the consolidated framework for implementation research (CFIR) framework (Damschroder et al., 2009) to summarize published evidence regarding opportunities and challenges to the integration of digital health in medical training curricula from the literature and the Expert Recommendations for Implementing Change (ERIC) (Powell et al., 2015) to inform policymakers on potential implementation strategies for successful integration.

## Methodology

### Theoretical framework

The lack of theoretical frameworks for conducting and performing implementation research hinders the generalizability of the study findings (Kirk et al., 2015). To facilitate generalizability, several implementation frameworks have been proposed, including the consolidated framework for implementation research (CFIR), which was developed by Damschroder and colleagues to provide a systematic understanding of the constructs that influence the implementation of interventions (Damschroder et al., 2009). The framework provides a systematic assessment of barriers and facilitators through its five main domains, namely, i) intervention characteristics, which is concerned with the new strategy or program being implemented; ii) the outer setting, which is concerned with the external features from the community, that can influence implementation; iii) the inner setting, which is concerned with the features (political, social, physical) of an organization in which the implementation process is effected; iv) the individual characteristics domain, which is concerned with the characteristics and roles of individuals involved in the intervention or the process of implementation; and v) the implementation process, which involves a set of activities aimed at achieving individual- and organizational-level use of the intervention as designed. CFIR has a track record of facilitating the identification and addressing of contextual factors that may facilitate or hinder the practicability, functionality, adaptability, adaptability, and scalability of well-intended interventions (Mugyenyi et al., 2023) and has been used in the identification of barriers and motivators for private hospitals’ engagement in TB care (Tumuhimbise and Musiimenta, 2021). Therefore, utilizing CFIR to explore opportunities and challenges in integrating digital health modules into medical curricula is paramount to guide future rethinking of integration approaches to similar interventions. To identify implementation strategies for the identified challenges, we used the expert recommendations for implementing change (ERIC), a compilation of 73 implementation strategies that were developed by a panel of experts in implementation science and practice to foster action-oriented research and overcome identified challenges (Powell et al., 2015).

### Scoping Review Methodology

The findings of this review were reported using the Preferred Reporting Items for Systematic Reviews and Meta-Analyses (PRISMA) extension for the scoping reviews checklist (Tricco et al., 2018). The presentation of review findings was guided by the Arksey and O’Malley scoping review methodology (Arksey and O’Malley, 2005). This methodology allows the inclusion of diverse study types and outlines six main steps to be followed while conducting a scoping review, namely, i) identifying the research question, ii) identifying relevant studies, iii) selecting studies, iv) charting data, v) collating, summarizing and reporting results, and vi) consulting (this step was not considered for this review). We purposively searched, identified, screened, and analyzed relevant studies that discussed the integration of digital health modules into the medical curriculum. There is no published protocol for this review.

### Identification of the Research Question

This study was guided by two main research questions: *i) what are the opportunities for integrating digital health training in medical curricula, and ii) what challenges are encountered during the integration of digital health training in medical curricula?* Therefore, the articles identified by the reviewers intended to address the questions above.

### Identification of Relevant Studies

We conducted a comprehensive review of the relevant literature in August 2024 to identify published studies that captured the current state of the art regarding the opportunities and challenges of integrating digital health training in medical curricula globally for inclusion. To identify these relevant studies, the Google Scholar, PubMed, and ScienceDirect bibliographic databases were used because of their high indexing capabilities for peer-reviewed articles. The authors reviewed articles published between 1st January 2014 and 31st August 2024 to understand the most recent literature about existing opportunities and challenges of digital health training integration in medical curricula. A combination of the following key search terms to denote ‘digital health training’, ‘challenges’, ‘opportunities’, and ‘Medical Curriculum’ was used in the bibliographical databases to identify the relevant articles.

Additionally, the bibliography/reference lists of the identified articles were also screened and reviewed for potential additional relevant studies. EndNote X7 (Thomson Reuters, Philadelphia, PA, USA) was used to manage and organize the search results obtained and to facilitate independent assessment among authors for inclusion on the basis of title, abstract, and full text.

### Study Selection

Studies were included if they were 1) peer reviewed, 2) full research papers available, 3) clearly described opportunities and/or challenges of integrating digital health training in medical curricula or the opportunities and challenges discussed in the methods section, 4) published between 1st January 2014 and 31st August 2024, or 5) available and published in the English language. Criteria 1) and 2) were considered to ensure the reporting of original research and high-quality work. Criteria 3) was included to ensure that the paper reported digital health training integration in medical curricula. Our definition of digital health training in medical curricular opportunities included advantages, benefits, facilitating factors, and motivators for integrating digital health training into curricula, while we defined challenges as difficulties, barriers, gaps, and problems encountered during the integration of digital health training in medical curricula. Criteria 4 was considered because in 2014, digital health interventions were more pronounced; therefore, the inclusion of research papers published before 2014 would imply the reporting of findings that were not recent regarding digital health training integration in medical training curricula. Studies were excluded if they did not report opportunities and/or challenges related to digital health training integration in medical curricula or if they were carried out before 2014 and if they were not relevant to the research questions of this review. All the studies were explicitly scrutinized to ensure that they reported opportunities and challenges; therefore, we did not include protocols; editorials; letters; position papers; or opinion pieces. We included all the relevant studies irrespective of the study design and geographical location.

### Charting of the Data

The authors reviewed titles and abstracts to identify relevant articles for final inclusion. The following characteristics were extracted from the eligible studies: author, study design/method/location, study objective, digital health module, opportunities, and challenges. All the extracted data are included in Table 1. All authors reviewed the articles at length for inclusion in the final analysis.

### Collating, summarizing, and reporting results

A series of iterative meetings, reviews, and discussions were held both virtually and physically with all the research team members to assess, analyze, and agree on the articles for final inclusion in this scoping review. The main characteristics of interest are tabulated in Table 1 to highlight the extraction of the parameters of interest from the identified studies. The structuring of the main findings was guided by the CFIR to extract relevant opportunities and challenges.

## Results

**Figure 1:**
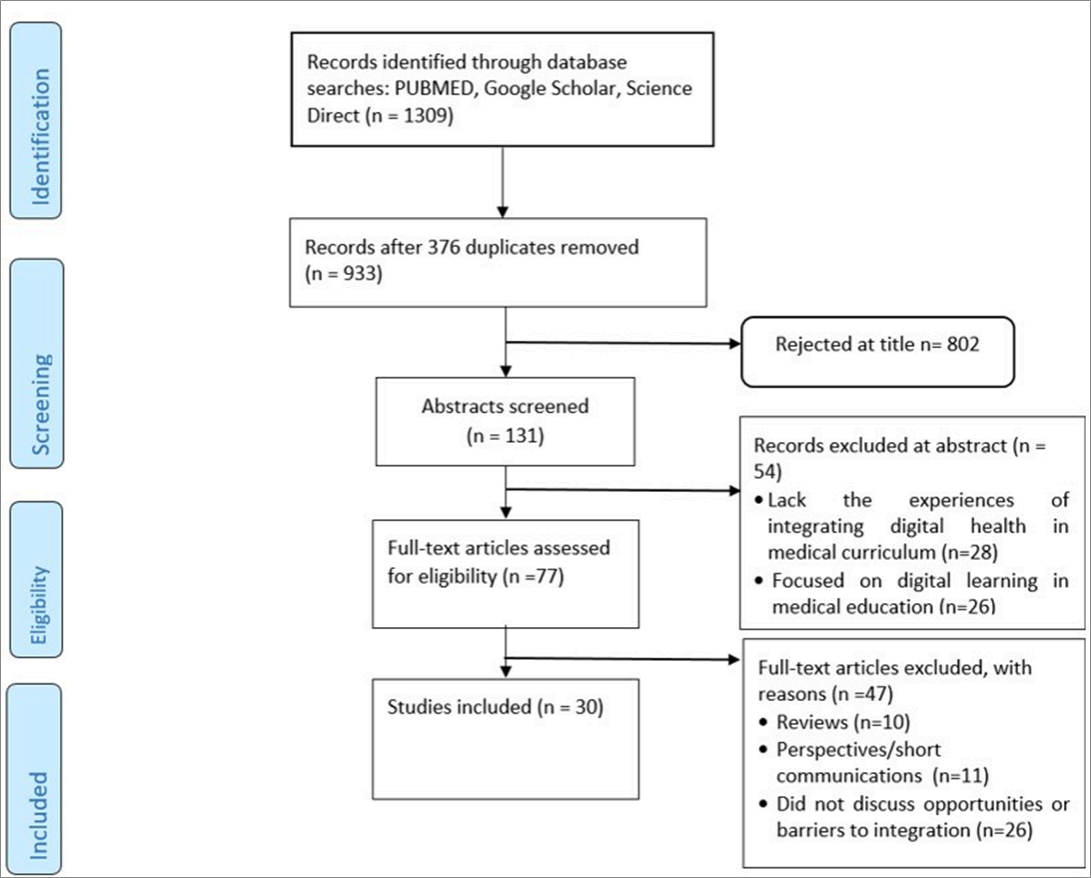
Flow diagram for the selected studies

Our initial database search identified 1309 articles, 376 of which were duplicates, as shown in Fig. 1 below. Eight hundred two articles were eliminated after screening the titles, and an additional 54 articles were removed from the abstracts. Forty-seven articles were excluded from the full-text review. Therefore, 30 studies were included in the analysis. The majority of the studies were conducted in Europe (60%, 18/30), 27%, 8/30 were conducted in Asia, with only 7%, 2/30 from North America, whereas both Africa and Australia accounted for only 3%, and 1/30, respectively, of the studies, as shown in Table 1.

Of the 39 constructs of the CFIR framework evaluated, 22 were assessed on the basis of the relevant themes from the data concerning the opportunities and challenges, as indicated in Table 2, whereas the remaining 17 did not yield themes of interest.

Opportunities arose from the relative advantage, adaptability, evidence strength, and quality, relative priority, tension for change, knowledge and beliefs about the intervention, self-efficacy, culture, and engaging constructs. These include the urgent need for healthcare digitalization, reshaping the daily work of healthcare professionals, preparing students for their future medical work by increasing their knowledge and attitudes, decreasing their doubts about eHealth technologies, and thus enhancing the quality of patients’ care.

On the other hand, challenges arose from complexity, design quality and packaging, external policies and incentives, cosmopolitanism, peer pressure, readiness for implementation, compatibility, available resources, engagement, implementation climate, knowledge and beliefs about the intervention, self-efficacy, executing, reflecting and evaluating, and engaging constructs. These include concerns of erosion of basic clinical assessment, fear of abandonment of a generalist approach to healthcare, loss of patient contact due to digitized medicine, lack of infrastructure and educational materials to operate digital health classes, the dense nature of the existing undergraduate medical curriculum, bureaucratic tendencies, lack of collaboration among medical schools, lack of standardization and clear policy guidelines, lack of clinically trained faculty with technical expertise to teach digital health, and seeing digitalization as a threat.

### Characteristics of the intervention

Six studies conducted in Germany, China, Europe, and Finland to introduce digital health as a curriculum module and identify undergraduate medical competencies in digital health and their suitable teaching methods highlighted the need to prepare medical students as early as possible in preparation for highly digitized future healthcare systems (Ma et al., 2023, Machleid et al., 2020, Poncette et al., 2020, Seemann et al., 2023, Veikkolainen et al., 2023, Alfallaj et al., 2022, Ejaz et al., 2022). This is aimed at enhancing the adoption of future digital health processes and technologies, training them in utilizing digital health applications (Alsahali, 2021), and preparing them for their perceived job qualifications to maintain their clinical responsibilities.

The integration of the digital health module in the medical curricula was reported to potentially decrease students’ doubts, reduce fears, change their attitudes and promote enthusiasm for digital health technologies (Machleid et al., 2020, Brockes et al., 2017, Darnell et al., 2023, Nitsche et al., 2023, Poncette et al., 2020). Additionally, it was reported to promote a culture of innovation, improve work efficiency among learners (Kröplin et al., 2024, Zainal et al., 2022), and facilitate access to quality care in remote and rural communities and reduce medical errors (Ejaz et al., 2022, Alsahali, 2021).

However, several studies have noted concerns about the erosion of basic clinical assessment skills due to overreliance on imaging, scanning, and laboratory results instead of physically examining patients (Zainal et al., 2022). A similar study reported concerns regarding depersonalization by digital technologies, where doctors may spend a considerable amount of time on their computers and do not maintain physical eye contact, which affects doctor-to-patient interaction and results in the fear of abandonment of a generalist approach to healthcare and the loss of patient contact due to digitized medicine (Zainal et al., 2022, Sorg et al., 2022). These have been noted to result in incorrect decisions, which raises ethical concerns (Nitsche et al., 2023, Ejaz et al., 2022).

### Inner setting

The urgent need to integrate digital health into the medical curriculum due to the growing use of digital technologies within health care has been reported. This is aimed at improving students’ ability to provide quality patient care, and to learn more about digital health concepts such as data protection, management, analysis, and AI in their medical courses to rationalize their intention to use digital tools as physicians (Lotrean and Sabo, 2023). (Alfallaj et al., 2022, Evbuomwan et al., 2020, Lotrean and Sabo, 2023, Machleid et al., 2020, Paré et al., 2022, Sakellari et al., 2024, Edirippulige et al., 2022, Ejaz et al., 2022). (Alsahali, 2021, Faihs et al., 2022, Farooq et al., 2024, Kröplin et al., 2024, Lotrean and Sabo, 2023, Ma et al., 2023, Zainal et al., 2023a, Edirippulige et al., 2022, Sorg et al., 2022).

However, this integration would require more evidence of the effectiveness of these digital technologies, a strong and integrated IT infrastructure in healthcare institutions (Zainal et al., 2023a), and centralized IT training to improve the current system of training (Walpole et al., 2016). Concerns regarding the lack of infrastructure in terms of the software and hardware necessary to use digital tools or platforms in digital medicine, IT insecurity (Walpole et al., 2016), a lack of backup systems in the event of system failure (Sorg et al., 2022) and a lack of access to the internet (Ejaz et al. 2022) have been raised as key challenges to the digitalization of medicine. This, in the long run, may hinder students’ ability to cope with the technical requirements of the program.

Nine studies have been conducted in Germany, China, Finland, Europe, Nigeria, and Australia (Poncette et al., 2020, Seemann et al., 2023, Ma et al., 2023, Veikkolainen et al., 2023, Machleid et al., 2020, Evbuomwan et al., 2020, Sorg et al., 2022, Faihs et al., 2022, Nitsche et al., 2023, Edirippulige et al., 2022), underscoring how digital health reshapes the future daily work of health care professionals by changing the way doctors and patients deal with each other and simplifying doctor consultations and ensuring 24/7 doctor access and medical on-the-spot support from paramedics (Gillissen et al., 2022).

Additionally, digital tools are described as key in facilitating the diagnosis, treatment, and rehabilitation of various diseases (Lotrean and Sabo, 2023) and supplementing traditional treatment and consultation (Brockes et al., 2017, Gillissen et al., 2022).

Several studies have reported the dense nature of the existing undergraduate medical curriculum (Zainal et al., 2023a, Machleid et al., 2020, Park et al., 2022) in comparison with the broad content of digital health (Park et al., 2022), which makes the integration of the new training module challenging. This means an increase in workload for both students and instructors (Alsahali, 2021, Gillissen et al., 2022).

Additionally, the lack of educational materials to operate digital health classes in China (Park et al., 2022), lack of digital health-related formats in medical education in Germany (Machleid et al., 2020), and lack of protective mechanisms in medical schools for experiential learning and experimentation in terms of safe and innovative spaces in Singapore and Canada (Zainal et al., 2023b, Paré et al., 2022, Raghunathan et al., 2023) have been reported as factors hindering students from practicing with digital health innovatively, which makes integration into medical curricula difficult.

Administratively, bureaucratic inertia, which makes it difficult to adjust the medical curriculum to incorporate new changes and requires considerable energy and resources to convince policymakers and medical school faculty, has been reported in Singapore. These efforts are counteracted by great resistance from individuals who are not well versed with modern technology (Zainal et al., 2023b).

### Outer setting

A lack of collaboration among medical schools due to different missions and friendly competition to produce competent medical graduates and a lack of standardization and clear policy guidelines regarding digital use in clinical practice were reported in Singapore as challenges hindering the integration of digital health in medical curricula (Zainal et al., 2023a). Additionally, the exponential growth of technological developments and rapid pace of technology advances leave educators with the responsibility of keeping up to date regarding current trends in digital health innovations (Zainal et al., 2022), making it challenging to train medical students in certain digital health technologies, yet they may not be applicable in the next few years.

### Characteristics of individuals

A pre-post test study carried out in Germany among students who participated in the transdisciplinary digital health curriculum at the University of Rostock reported an increase in knowledge of digital health competence and better overall coverage of digital health learning objectives among clinical students (Kröplin et al., 2024). Additionally, an evaluation study of clinical telemedicine/e-health module integration in the curriculum among medical students at the University of Zurich reported increased clarity about the need for telemedicine, increased overall satisfaction and understanding of telemedicine, and increased willingness to use telemedicine for chronically ill and elderly patients (Brockes et al., 2017). Another quantitative study (Darnell et al., 2023) at the University of Southern California that integrated case conference series reported an increase in students’ knowledge of digital competencies, familiarity, and comfort with smart pills; digital therapeutics; health and wellness apps for smart devices; and telehealth and improved their perception that digital health is an important aspect of patient care.

Positive attitudes among medical students and lecturers toward digitization and incorporating digital health into the medical curriculum have been documented as key opportunities for digital health training integration. Two studies conducted in Germany and the Netherlands (Machleid et al., 2020, Vossen et al., 2020) reported that medical students had a positive attitude toward this integration and were willing to play a central and active role as mediators of digital health literacy to patients. Additionally, positive attitudes toward the digitization of healthcare, the use of digital tools in different domains within the medical field, and the intention to use digital tools as physicians have also been reported in Germany and Romania (Lotrean and Sabo, 2023, Nitsche et al., 2023). This could be due to the perceived belief among students about digital health (Alsahali, 2021) and that the future is digital (Evbuomwan et al., 2020) in anticipation that medicine will be fundamentally changed by new digital opportunities in the next few years (Faihs et al., 2022).

However, the lack of preparedness among students to address digital challenges in their future profession and to take advantage of technological developments within the medical field (Brockes et al., 2017, Park et al., 2022, Raghunathan et al., 2023, Sorg et al., 2022, Vossen et al., 2020, Faihs et al., 2022) and the lack of skills to use these digital tools (Darnell et al., 2023, Machleid et al., 2020, Walpole et al., 2016, Zainal et al., 2023a, Faihs et al., 2022) Casa et al., 2021), difficulties in convincing doctors to use digital services and apps (Faihs et al., 2022, Gillissen et al., 2022, Poncette et al., 2020), are described as key challenges that may counteract the future implementation of digital technologies. This could be attributed to the lack of clinically trained faculty with technical expertise to teach digital health and digital health content creation (Alfallaj et al., 2022, Walpole et al., 2016). This makes it difficult for students to understand different terminologies such as data protection and artificial intelligence used in digital health (Walpole et al., 2016; Brockes et al., 2017; Gillissen et al., 2022; Park et al., 2022), which subsequently results in poor digital health skills (Machleid et al., 2020; Darnell et al., 2023; Evbuomwan et al., 2020; Raghunathan et al., 2023; Zainal et al., 2023a; Sorg et al., 2022; Edirippulige et al., 2022) (Casa et al., 2020; negative attitudes toward digital health (Sorg et al., 2022; Edirippulige et al., 2022); and a lack of awareness of the need for digital health (Park et al., 2022). Therefore, early and increased integration of digital medicine topics can potentially bridge this gap.

A study in Saudi Arabia noted that digital health requires more mental effort (Alsahali, 2021), and even recent advances in digital health, such as the use of artificial intelligence, viewed more as encumbrances than as useful assistance (Gillissen et al., 2022).

### Implementation process

Ten studies reported the urgent need for healthcare digitalization (Veikkolainen et al., 2023, Vossen et al., 2020, Baumgartner et al., 2022, Darnell et al., 2023, Kröplin et al., 2024, Ma et al., 2023, Nitsche et al., 2023, Poncette et al., 2020, Zainal et al., 2023a, Edirippulige et al., 2022) as a continuous process to improve the analysis of patient data to make evidence/based data-driven clinical care (Poncette et al., 2020). This digitization should be accepted by those who perceive it as relevant to their work (Kröplin et al., 2024). This is meant to act as an accessory tool to improve their performance, save time, make their work easier (Gillissen et al., 2022), and improve patient safety within the operations theatre (Baumgartner et al., 2022), not as a threat to their jobs (Nitsche et al., 2023). However, digitalization is still seen as a threat, especially concerning the patient‒physician relationship (Baumgartner et al., 2022). A study by Gillissen and colleagues reported that unreliable and noncertified internet sources that patients always receive may confuse the patient–doctor relationship, especially when the physician disagrees with the patient’s medical inquiry (Gillissen et al., 2022).

Six studies reported that digital health is not sufficiently integrated into the current undergraduate curriculum in Germany, Australia, Saudi Arabia, Singapore, or the United Kingdom (Seemann et al., 2023, Raghunathan et al., 2023, Alfallaj et al., 2022, Kröplin et al., 2024, Park et al., 2022, Walpole et al., 2016). This is due to a lack of formal teaching where digital health is integrated into other topics, while another study in Singapore reported inconsistent opening of digital health classes, and another study in Saudi Arabia reported high costs related to the integration of digital health in medical curricula (Alfallaj et al., 2022).

## Discussion

### Summary of evidence

Guided by the CFIR framework, our review sought to summarize published evidence regarding opportunities for and barriers to the integration of digital health education in medical curricula from the literature to inform policymakers on factors that should be considered for future integration. Several opportunities for digital health integration in medical training identified by this scoping review include the urgent need for healthcare digitalization, reshaping the future daily work of healthcare professionals, preparing students for their future medical work by increasing their knowledge and attitudes, and decreasing their doubts about digital health, thus enhancing their quality of patient care. In contrast, concerns of erosion of basic clinical assessment, fear of abandonment of a generalist approach to healthcare, loss of patient contact due to digitized medicine, lack of infrastructure and educational materials to operate digital health classes, the dense nature of the existing undergraduate medical curriculum, bureaucratic tendencies, lack of collaboration among medical schools, lack of standardization and clear policy guidelines, lack of clinically trained faculty with technical expertise to teach digital health, and seeing digitalization as a threat were identified as barriers to digital health integration in medical training.

In his remarks during the unveiling of the WHO’s Smart AI Resource Assistant for Health (SARAH), a generative AI for understanding the risk factors for the leading causes of death in the world, Dr. Tedros Adhanom Ghebreyesus, the director-general of the WHO, noted that “the future of health is digital” (WHO, 2024). This cannot be disputed given that information communication technologies (ICTs) have been underscored by the 2030 global agenda for sustainable development as key in accelerating human progress and bridging the digital divide gap (Assembly, 2015). The advent of digital technologies such as wearable devices, computerized clinical decision support systems, and telemedicine, which are frequently used in healthcare settings, is a testament in which digital health is a larger component of the overall healthcare system and plays an important role in improving the technical performance and quality of delivered care (Borges do Nascimento and Abdulazeem, 2023). The use of these digital tools in clinical practice seems inevitable given their widespread preference among users. This, therefore, calls for mechanisms that can carefully consider addressing the identified challenges and leveraging the identified opportunities to help introduce digital health to these future users as part of their formal and informal education. This is aimed at the development of a critical digital health mindset with openness to innovation, and the ability to assess the ever-changing health technologies needed to translate research into clinical care should be prioritized (Poncette et al 2020).

The desire to digitize health to allow seamless collection and use of data to facilitate evidence-based medicine for enhancing patient-centered care requires building the capacity of future users to facilitate adoption and usability. Therefore, training users as early as possible fosters intervention ownership and acts as a mindset and attitude reset for those learners with negative attitudes/low perceptions of the usefulness of digital health. Several physicians have already lamented about the challenges of preparing family medicine residents for the digital era. In his commentary, Dr. Rashad Bhyat noted that the next generation of physicians “will be the most digitally savvy to date” and advocated for tools and resources that can facilitate optimal utilization among these physicians to benefit their patients and facilitate better clinical experience (Bhyat, 2019). Training clinicians in the practical use of these technologies as early as possible has long been overdue (Chandrashekar, 2019).

The already dense nature of the medical curriculum makes it challenging for planners to add more content such as digital health (Zainal et al., 2022), which may be attributed to faculty resistance to change toward an integrated curriculum (Hafeez et al., 2021, Price and Regehr, 2022). Truthfully, the pace at which the world is advancing toward digitization is proof that traditional medical practice will not remain the same. The National Academy of Medicine outlined key areas where digital health is currently being applied in almost every aspect of medicine, including health information (digital records and dashboard), knowledge generation (epigenetics, epidemiologic modeling), knowledge integrators (predictive analytics and decision aids), personal health devices, telemedicine, diagnostics, imaging for pinpoint assessment and interpretation, dose use and monitoring, implantable devices, and robotics for surgical practice, among others. Therefore, burying our heads in the sand even with these evident projections and the high penetration rates of digital technologies and how they are at the forefront of revolutionizing healthcare would be a great inconsideration of our time. In his book “Future Shock”, Alvin Toffler declared that “The illiterate of the twenty-first century will not be those who cannot read and write, but those who cannot learn, unlearn, and relearn” (Toffler, 1970). It is, therefore, time we learned, unlearnt and relearnt through rethinking and reimagining the delivery of clinical practice in the 21st century.

The existing knowledge gap regarding the importance of these digital health interventions and the lack of perceived usefulness of digital health courses (Park et al., 2022) affects the overall success of the implementation of these well-intended interventions. This is exacerbated by the negative attitudes toward digital health (Sorg et al., 2022, Edirippulige et al., 2022) and the view of digitization as a threat to patient‒physician relationships (Baumgartner et al., 2021), which have been noted as a significant challenge to digital health integration in medical curricula. Studies have shown that 49% of all Android apps downloaded are deleted before one month of usage (Freer, 2023). It may be true that some usability problems arise, but a lack of understanding of the purpose of such interventions may be a contributing factor. Institutional training mechanisms such as sensitization programs in the form of webinars underscoring the role of digital health and debunking myths about digital health may play an important role in addressing these challenges and setting a stage for smooth integration and utility.

Whereas some of the identified challenges are indeed external, organizational, and procedural, such as a lack of policy guidelines, standardization, and a lack of trained staff, which may be beyond the control of individuals, devising means for lessening their negative impact on digital health integration is crucial. This requires concerted efforts and political commitment from governments and international organizations to guide the importance of this integration toward the global digital health agenda 2030 and the necessary moral and financial support toward establishing standards, policy frameworks, and guidelines for this integration in medical curricula. Alternatively, longitudinal studies assessing the outcomes of the intervention by comparing students enrolled in a medical curriculum that utilized digital health in their medical practices may offer more reasons for institutions to weigh the benefits and risks of churning digitally competent medical workers.

Using the ERIC (Powell et al., 2015), we identified 16 implementation strategies, as indicated in Table 3, that can be used to mitigate the identified challenges. These include providing consolidated funds tailored to support integration and technical assistance during the learning process; setting up a dedicated digital health infrastructure to support the experiential learning process, starting with both elective-based and audited modular approaches; raising awareness and educating key stakeholders about the potential of digital health and its integration in medical curricula; involving key stakeholders as early as possible during planning and curriculum design; establishing a clear roadmap toward the formalization of digital health module teaching in institutions; partnering with other institutions for benchmarking, knowledge, and training resource sharing; establishing clear monitoring processes for better outcomes; distributing key digital health educational materials; identifying passionate, vocal individuals, early adopters in institutions to advocate for integration and overcoming resistance to change; benchmarking on the expertise of governing structures at institutions for guidance; and recruiting new staff with specific training in digital health are key implementation strategies that can mitigate the identified challenges.

Although this review has shown that there has been global progress toward the incorporation of digital health training in medical curricula, their integration in Africa is still minimal. The evidence shows that significant strides toward digital adoption are being made, as more than 47 countries have already developed digital health strategies for building and strengthening resilient health systems (Victor et al., 2023). Although this approach is commendable, progress toward the WHO’s advocacy for ensuring digital literacy integration in formal and informal education curricula by African countries remains rare compared with that of European nations and North America. This indicates slow progress toward having all healthcare workers prepare to deploy and use digital health tools in their daily work. Future implementers need to envisage mechanisms for integrating digital health into medical curricula.

### Implications for policy and implementers

Training users as early as possible fosters intervention ownership and acts as a mindset and attitude reset for those learners with negative attitudes/low perceptions of the usefulness of digital health. This requires a systematic approach to identifying bottlenecks and capitalizing on the available opportunities to ensure smooth integration at early entry points, such as medical curricula. The question left to all of us is whether to let these bottlenecks remain and maintain the way things are even when it is evident that digital health is here to stay or prepare and brace ourselves and our institutions by garnering the necessary support toward this digital future.

### Strengths and Limitations

To the best of our knowledge, our review is the first to synthesize evidence for policymakers, future researchers, and implementers about contextual multilevel bottlenecks that should be addressed to realize opportunities for digital health integration in medical curricula a. If these opportunities are leveraged, they can improve digital health integration outcomes in medical curricula. Failure to address them might compromise the quality of digital integration. Furthermore, our review was based on the key implementation science principles of identifying evidence-practice gaps and performing data analysis and extraction on the basis of a theoretical implementation framework.

However, our study was not without limitations. First, our review contributes to the understanding of digital health training integration by reporting several studies that have integrated digital health training as a part of the curriculum and other studies that only report perceived experiences, attitudes, and perceptions. We believe that several other institutions have integrated digital health training but have not published their experiences in peer-reviewed journals (Aungst and Patel, 2020, Tudor Car et al., 2021). However, a search of their institutional databases can yield better outcomes. Therefore, it is important to note that there could be institutions that have integrated digital health into their medical curriculum but have not published their experiences but were not included in the final review. Additionally, we did not include papers that focused only on assessing digital competencies if they did not report opportunities and challenges or studies that reported factors related to the use of e-learning or digital education tools.

## Conclusion

The integration of digital health training in medical curricula cannot be successful if bottlenecks are not identified and if mechanisms are put in place to address them. Global progress toward integrating digital health literacy in formal medical training curricula remains slow. Thus, there is a need for concerted efforts and political commitment from governments and international organizations to guide the importance of this integration toward the 2020–2025 global digital health agenda and the need for moral and financial support to establish standards, policy frameworks, and guidelines for this integration in medical training curricula. Therefore, the successful integration of digital health in medical training requires careful efforts to address these existing challenges and leverage these opportunities.

## Figures and Tables

**Figure 2:**
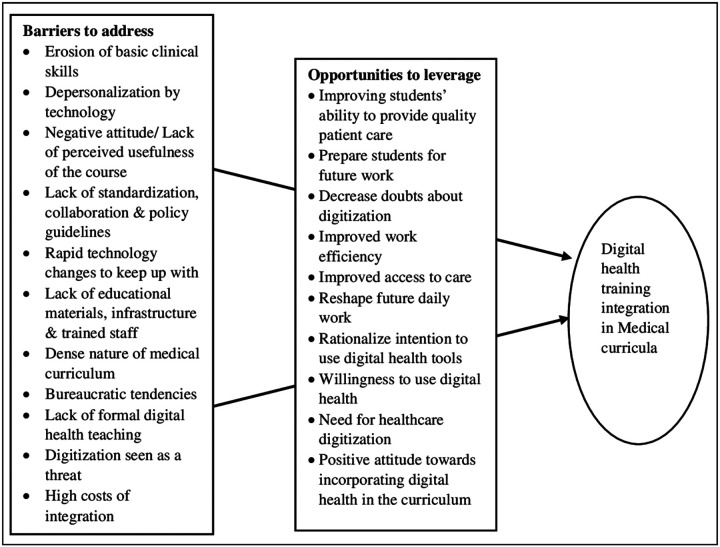
Challenges to address and opportunities to leverage toward digital health training integration in Medical curricula

**Table 2:** CFIR Constructs and their related opportunities or challenges to digital health integration in medical education curricula

CFIR domain	CFIR construct	Opportunity or challenge	Explanation for opportunities and challenges	
Intervention Characteristics	Relative advantage	Opportunity	Improving students’ ability to provide quality patient care	
	Relative advantage	Opportunity	Preparing students for their future medical work	
	Adaptability	Opportunity	Decrease students’ doubts about digital Health technologies	
	Evidence strength and quality	Opportunity	Improve work efficiency among learners	
	Evidence strength and quality	Opportunity	Improve access to care in underserved communities and the quality of care in remote and rural communities and reduce medical errors	
	Complexity	Challenge	Erosion of basic clinical assessment skills	
	Design quality and packaging	Challenge	Depersonalization by technology	
	External policies and incentives	Challenge	Lack of standardization	
Outer setting	Cosmopolitanism Peer pressure	Challenge	Lack of collaboration among medical schools due to different mission and friendly competition to produce competent medical graduates	
	External policies and incentives	Challenge	Lack of clear policy guidelines for clinical practice	
	Peer pressure	Challenge	The exponential growth of technological developments and rapid pace of technology advances	
	Relative priority	Opportunity	Improving students’ ability to provide quality patient care and to satisfy their desire to learn more about digital health concepts	
Inner setting	Tension for change	Opportunity	Rationalize their intention to use digital tools as physicians	
	Culture	Opportunity	Reshaping the future daily work of health care professionals	
	Readiness for implementation	Challenge	Lack of educational materials to operate digital health classes	
	Compatibility	Challenge	Dense nature of the existing undergraduate medical curriculum increase in workload for both students and instructors
	Available resources	Challenge	Lack of Infrastructure (necessary software and hardware, internet) to use respective digital tools or platforms in digital medicine
	Engagement	Challenge	Bureaucratic tendencies
	Implementation climate	Challenge	Lack of protective mechanisms in medical schools for experiential learning and experimentation in terms of safe and innovative spaces
	Knowledge and beliefs about the intervention	Opportunity	Improved perception that digital health is an important aspect of patient care
Individual Characteristics	Knowledge and beliefs about the intervention	Opportunity	Increase in knowledge in digital health competence
	Self-efficacy	Opportunity	Increased overall satisfaction, understanding and willingness to use digital health for chronically ill and elderly patients
	Knowledge and beliefs about the intervention	Opportunity	Positive attitudes among medical students and lecturers toward digitization and incorporating digital health into the medical curriculum
		Challenge	Lack of clinically trained faculty with technical expertise to teach digital health and digital health content creation
		Challenge	Negative attitude toward digital health (Difficulties in convincing doctors to use digital services and apps)
		Challenge	Lack of awareness of the need for digital health (perceived usefulness of the courses)
	Self-efficacy	Challenge	Lack of preparedness among students to address digital challenges in their future profession and to take advantage of the technological developments within the medical field
		Challenge	Digital health requires more mental effort
	Engaging	Opportunity	Urgent need for health care digitalization
	Executing	Challenge	High costs related to the integration of digital health in medical curricular
Implementation Process	Reflecting and evaluating	Challenge	Digitalization seen as a threat to the patient-physician relationship
	Engaging	Challenge	Digital health is not sufficiently integrated in the current undergraduate curricular
		Challenge	Lack of formal teaching

**Table 3:** Matching the challenges with implementation strategies per the ERIC framework to guide smooth integration

Challenges	ERIC Implementation Strategies	Application of the strategies
High costs of integration	Access new funding	Provision of consolidated funds tailored to supporting the integration
Digital health requires more mental effort	Centralize technical assistance	Provide technical assistance to learners during the process of learning
Lack of Infrastructure (necessary software and hardware, internet) to use respective digital tools or platforms in digital medicine	Change physical structure and equipment	Intentionally set up the digital health infrastructure (dedicated digital health labs, provide dedicated internet and digital resources) to support experiential learning process
Lack of protective mechanisms in medical schools for experiential learning and experimentation in terms of safe and innovative spaces		
Dense nature of the existing undergraduate medical curriculum increase in workload for both students and instructors	Conduct cyclical small tests of change	Adopt an elective based modular approach, or an audited digital health module, evaluate the performance outcomes and areas of refinement
Digitization seen as a threat	Conduct educational meetings and outreach visits	Aimed at raising awareness and educating key stakeholders about the role and potential of digital health and its integration in medical curricular
The exponential growth of technological developments and rapid pace of technology advances		
Depersonalization by technology		
Erosion of basic clinical assessment skills		
Bureaucratic tendencies	Conduct local consensus discussions	Involve key stakeholders (university leaders, top management, professional bodies) as early as possible during planning and curriculum design
Lack of formal digital health teaching	Develop a formal implementation blueprint	Establish a clear roadmap toward formalization of digital health module teaching
Lack of collaboration among medical schools due to different mission and friendly competition to produce competent medical graduates	Develop academic partnerships	Partnering with sister institutions for benchmarking, knowledge, and training resources sharing
Lack of standardization	Develop and organize quality monitoring systems	Standardize the process of teaching at institutions by establishing clear monitoring processes for better outcomes
Lack of educational materials to operate digital health classes	Distribute educational materials	Development and distribution of key digital health educational materials including guidelines, reading materials
Negative attitude	Identify and prepare champions	Identify passionate, vocal individuals in institutions to advocate for integration and overcome resistance to change
Lack of perceived usefulness of the course	Identify early adopters	Early adopters within institutions for other learners to learn from their experiences
Lack of clear policy guidelines for clinical practice	Involve executive boards	Benchmark on the expertise of governing structures at institutions to offer guidance and foster policies regarding digital health integration within their institutions
	Use advisory boards and workgroups	Additionally, these stakeholders may play a role in providing input and advice on the digital health module integration in medical curricular roadmap
Lack of preparedness among students to address digital challenges in their future profession and to take advantage of the technological developments within the medical field	Prepare consumers to be active participants	Equip learners to actively ask questions and seek for guidance during learning process about the importance of digital health in their future profession.
Lack of trained staff	Recruit, designate, and train for leadership	Intentionally allocate adequate funding to facilitate the recruitment of key staff with specific training in digital health

## Data Availability

The datasets used and/or analyzed during the current study are available from the corresponding author upon reasonable request.
